# Deciphering the Role of Functional Ion Channels in Cancer Stem Cells (CSCs) and Their Therapeutic Implications

**DOI:** 10.3390/ijms26157595

**Published:** 2025-08-06

**Authors:** Krishna Samanta, Gali Sri Venkata Sai Rishma Reddy, Neeraj Kumar Sharma, Pulak Kar

**Affiliations:** 1 Department of Biotechnology, Koneru Lakshmaiah Education Foundation, Vaddeswaram, Guntur 522302, India; 2 Department of Biological Sciences, SRM University-AP, Amaravati 522240, India; sairishmareddy_gali@srmap.edu.in; 3 Department of Computer Science Engineering, SRM University-AP, Amaravati 522240, India; neerajkumar.s@srmap.edu.in

**Keywords:** cancer stem cell, ion channels, cellular signalling, tumour microenvironment, ion channel-targeted therapy, cancer therapeutics

## Abstract

Despite advances in medicine, cancer remains one of the foremost global health concerns. Conventional treatments like surgery, radiotherapy, and chemotherapy have advanced with the emergence of targeted and immunotherapy approaches. However, therapeutic resistance and relapse remain major barriers to long-term success in cancer treatment, often driven by cancer stem cells (CSCs). These rare, resilient cells can survive therapy and drive tumour regrowth, urging deeper investigation into the mechanisms underlying their persistence. CSCs express ion channels typical of excitable tissues, which, beyond electrophysiology, critically regulate CSC fate. However, the underlying regulatory mechanisms of these channels in CSCs remain largely unexplored and poorly understood. Nevertheless, the therapeutic potential of targeting CSC ion channels is immense, as it offers a powerful strategy to disrupt vital signalling pathways involved in numerous pathological conditions. In this review, we explore the diverse repertoire of ion channels expressed in CSCs and highlight recent mechanistic insights into how these channels modulate CSC behaviours, dynamics, and functions. We present a concise overview of ion channel-mediated CSC regulation, emphasizing their potential as novel diagnostic markers and therapeutic targets, and identifying key areas for future research.

## 1. Introduction

Cancer remains one of the leading causes of mortality worldwide, driven in part by a subpopulation of tumour-initiating cells known as cancer stem cells (CSCs), which play a critical role in tumour initiation, progression, and resistance to therapy [1]. While ongoing innovations have significantly improved cancer treatment, urgent attention is needed to address the rising burden. Fundamental research remains critical, especially in understanding cancer biology at its root. Among the emerging areas, CSCs have garnered significant attention for their role in therapy resistance, tumour initiation, and relapse [2,3,4,5,6].

Normal stem cells are undifferentiated or partially differentiated cells capable of self-renewal and specialization [7,8]. In mammals, key types include embryonic stem cells (ESCs) from the blastocyst and adult stem cells (ASCs) found in tissues like bone marrow and liver, which remain mostly dormant but activate for tissue repair [7]. Induced pluripotent stem cells (iPSCs), generated by reprogramming adult cells with factors such as Oct4 and Sox2, mimic ESC behaviour [7]. A distinct population, CSCs, has been identified in various tumours [7,8]. CSCs, also known as tumour-initiating cells, are a small but aggressive subpopulation within tumours [8]. CSCs can arise either from normal tissue-resident stem cells or from differentiated cancer cells that acquire stem-like traits, often influenced by signals from the tumour microenvironment [8]. The concept of CSCs, first proposed in the late 1990s, has since become a focal point of intense investigation [9,10]. Over the past decades, extensive research has sought to reveal their roles in cancer biology [11]. CSCs have been identified and extensively characterized across a wide spectrum of tumour types [11]. These cells are identified by specific surface and intracellular markers such as CD44, CD133, ALDH1, and EpCAM [8]. CSCs have been identified across numerous cancer types such as breast, colon, lung, prostate, liver, melanoma, leukemia, head and neck, ovarian, pancreatic, and brain tumours [12]. Several key developmental pathways, including Notch, Hedgehog, and Wnt/β-catenin, regulate CSC behaviour [8]. These cells possess hallmark properties such as self-renewal, multilineage differentiation, and the unique ability to initiate and sustain tumour growth [13]. Importantly, CSCs are also endowed with a high proliferative potential, enabling them to drive tumour progression and heterogeneity. Due to these intrinsic capabilities, CSCs are increasingly recognized as the root cause of tumour recurrence and metastasis [11,14]. Even after aggressive clinical interventions such as surgical resection, radiotherapy, and chemotherapy, a small population of CSCs often survives. These residual cells can evade conventional therapies, regenerate the tumour microenvironment, and ultimately lead to disease relapse [11]. Consequently, many patients experience tumour recurrence and metastasis within a few years post treatment, emphasizing the critical role of CSCs in cancer persistence and poor long-term prognosis [5]. While CSCs share several characteristics with normal stem cells, such as the capacity for self-renewal and differentiation, they are distinguished by aberrant signalling cascades and a pronounced resistance to conventional therapies [15]. Among the diverse molecular regulators implicated in the maintenance and plasticity of CSCs, ion channels have recently emerged as fundamental modulators [16].

Ion channels, traditionally studied in the context of excitable tissues, are transmembrane proteins that regulate the selective movement of vital ions—such as calcium (Ca^2+^), potassium (K^+^), sodium (Na^+^), and chloride (Cl^−^)—across cellular membranes [17]. Beyond their classical roles in maintaining membrane potential and cellular excitability, ion channels profoundly influence crucial biological processes including cell proliferation, migration, apoptosis, and stemness-related signalling pathways [18]. Recent findings suggest that the dysregulation of specific ion channel families play a crucial role in maintaining the CSC phenotype, supporting their survival, invasiveness, and resistance to therapy [16]. Therefore, it is essential to gain a clear understanding of the ion channel expression profiles in CSCs and their regulatory roles in CSC fate and function, as this knowledge could pave the way for future research aimed at effectively combating cancer. Although a few reviews have discussed ion channels in CSCs, most focus on individual channel types. To the best of our knowledge, this is the first comprehensive review that encompasses a broad spectrum of ion channels expressed across various CSC types and their associated functions. We explore the wide range of ion channels found in CSCs and shed light on recent discoveries regarding their roles in controlling CSC behaviour, functional properties, and cellular dynamics. Special emphasis is placed on acid-sensing ion channels (ASICs) and aquaporin (AQP) channels, which, despite their significant roles in regulating CSC fate and function, have not been comprehensively reviewed until now.

## 2. Ion Channel Expression Profiles and Their Functions in CSCs

**Figure 1:**
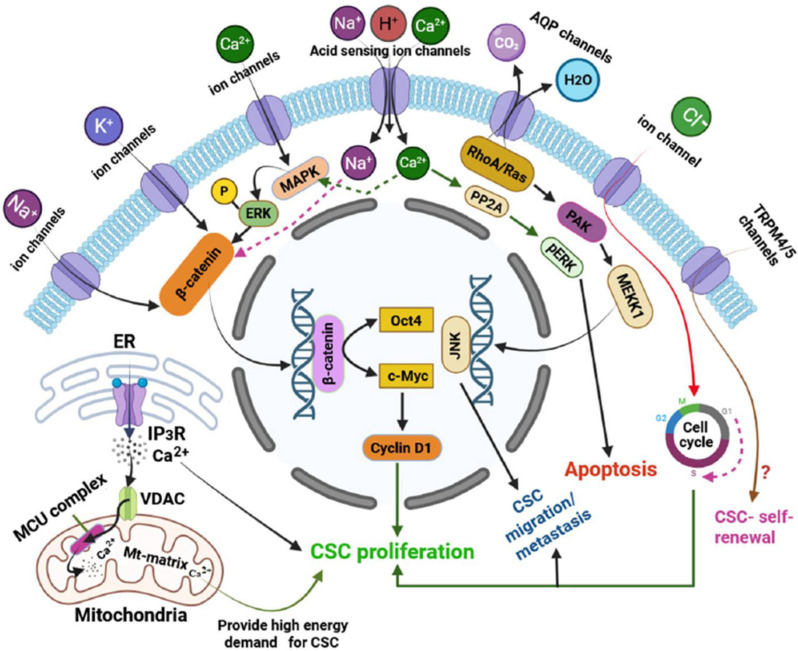
Key ion channels and their roles in regulating cancer stem cell (CSC) stemness and survival.

A diverse array of functional ion channels has been identified across various types of CSCs, exhibiting significant heterogeneity. CSCs exhibit a unique ion channel expression profile that distinguishes them from bulk tumour cells and normal stem cells. These ion channels span various classes, each contributing to distinct aspects of CSC biology. Through an extensive PubMed-based literature search focusing on ion channels in CSCs, we have compiled a comprehensive list of these channels, summarized in Table 1, deciphering their functional relevance and potential as therapeutic targets (Table 1 and Figure 1). The following section details specific ion channels and their involvement in CSC function.

This figure illustrates major plasma membrane- and organelle-associated ion channels that influence CSC properties by modulating signalling pathways, ion homeostasis, and cellular functions. Sodium (Na^+^) and potassium (K^+^) channels activate intracellular cascades converging on the β-catenin pathway, resulting in the upregulation of Oct4 and c-Myc, which drive CSC proliferation. Calcium (Ca^2+^) influx activates the MAPK/ERK pathway, which further enhances β-catenin-mediated transcription and CSC expansion [11]. Acid-sensing ion channels (ASICs) have dual roles: Ca^2+^ influx via ASIC1 induces PP2A-dependent apoptosis, while Na^+^ entry through ASICs may support CSC survival via β-catenin activation [11,73]. Aquaporins (AQPs) promote CSC migration and metastasis through RhoA/Ras-JNK signalling [74], and CLIC1 supports proliferation by facilitating G1-to-S phase cell cycle progression [50]. TRPM4 and TRPM5, although Ca^2+^-impermeable, are associated with the maintenance of stemness, though their precise roles remain to be clarified [33]. The figure also highlights intracellular channels such as mitochondrial voltage-dependent anion channels (VDACs), which coordinate with endoplasmic reticulum (ER)-based IP_3_ receptors to import Ca^2+^ into mitochondria via the mitochondrial calcium uniporter (MCU), thereby enhancing mitochondrial energy production required for CSC function [3,75]. A portion of this ER-released Ca^2+^ also promotes CSC proliferation and stemness directly in the cytoplasm [3]. In the figure, solid arrows represent the activation of signalling pathways or ion transport, while dashed arrows indicate putative signalling routes and their downstream effects. All ion channels and associated mechanisms are described in detail in the main text.

## 3. Calcium Ion Channels and Their Functions

Intracellular calcium dynamics play a key role in CSCs functions with multiple calcium channels—including store-operated calcium entry (SOCE) channels, voltage-gated calcium channels (VGCCs), transient receptor potential (TRP) channels, calcium release channels (CRCs), and mechanosensitive channels [3,19,20,21,22,23,24,25,26,27,36,39,40,41].

### 3.1. SOCE Channels and Their Functions

SOCE is a key mechanism for calcium influx, activated by ER calcium depletion via PLC and IP3 signalling [8]. It involves STIM proteins (ER Ca^2+^ sensors) and Orai channels in the plasma membrane. Ca^2+^ release-activated Ca^2+^ channels or CRAC channels, mainly formed by Orai proteins, mediate highly selective Ca^2+^ entry to bombard a specific downstream target [8,20,21,22,23]. Variants like Orai1α and Orai1β show distinct activation patterns. Dysregulation of SOCE components can disrupt calcium homeostasis, promoting tumour growth, poor prognosis, and drug resistance [8]. Beyond SOCE, Orai1 also supports alternative calcium entry pathways through interactions with secretory pathway Ca^2+^-ATPase 2 (SPCA2), small-conductance calcium-activated potassium channels (SK3 or KCa2.3), and Kv10.1 channels. These interactions can sustain calcium entry and promote CSC survival and migration [8]. Using knockdown and rescue experiments in MDA-MB-231 cells, Jardin et al. [21] showed that Orai1 is crucial for spheroid formation and self-renewal in human epidermal growth factor receptor 2-positive (HER2+) and triple-negative breast cancer stem cells (BCSCs), but not in estrogen receptor-positive (ER+) BCSCs or BSCs [21]. Both Orai1α and Orai1β equally support SOCE, COX activation, and mammosphere formation in BCSCs [21]. Orai1 interacts with SPCA2 to drive glycolysis-dependent growth in BCSC-like cells via store-independent calcium entry (SICE), while Orai3 and STIM1 promote glycolysis-independent SOCE [19]. In acute myeloid leukemia (AML), Orai1–SOCE correlates with leukemic stem cell (LSC) proportion and drug resistance, indicating its utility as a biomarker [20]. Orai3 is enriched in CSCs and promotes stemness via the Orai3–ID1 (Orai3 inhibitor of DNA binding 1) axis in oral/oropharyngeal squamous cell carcinoma (OSCC) [24]. Terrié et al. [25] demonstrated that SOC entry supports glioblastoma stem cell (GSC) maintenance, likely through CaMKII and NFAT activation, highlighting calcium’s central role in stemness and tumour progression [25]. However, the precise contribution of SOC in CSCs remains to be fully defined.

### 3.2. TRP Channels and Their Functions

TRP ion channels, well-known for their roles in sensing physical and chemical stimuli, are increasingly recognized as important regulators of stem cell behaviour, including CSCs [26]. Several TRP family members including TRPC1/3/6, TRPV1/2/4/6, and TRPM7/8 are dysregulated during cancer progression, contributing to diverse oncogenic processes [28,29].

TRPA1 and TRPV1 are modulated by ERK signalling and transcription factor Runx1/Aml1 isoforms, influencing glioblastoma stem cell (GSC) differentiation and survival [27]. TRPC3 is frequently overexpressed in triple-negative breast cancer (TNBC), where it drives proliferation and chemoresistance via the TRPC3–RASA4–MAPK (TRP3-Ras GTPase-activating protein 4-MAPK) axis. Additionally, TRPC3 promotes NF-κB activation in cancer-associated fibroblasts, thereby enhancing tumour growth [30]. In breast cancer, TRPV4 and TRPV6 are upregulated and facilitate calcium influx, which supports cell proliferation, angiogenesis, and tumour progression [31].

TRPM7 plays a particularly versatile role in cancer biology. It promotes metastasis in non-small cell lung cancer (NSCLC) by enhancing O-GlcNAcylation and stabilizing key oncogenic proteins such as c-Myc and caveolin-1 [28]. In lung CSCs, elevated TRPM7 expression is associated with increased levels of stemness and metastatic markers including SOX2, KLF4, CD133, Hsp90α, uPA, and MMP2 [29]. Therapeutically, TRPM7 has emerged as a promising target [28,29]. Oridonin, a bioactive compound from *Rabdosia rubescens*, suppresses bladder cancer growth by targeting TRPM7 and inhibiting the ERK/AKT signalling pathway [32]. Interestingly, TRPM7 is unique among TRP channels in containing both an ion channel and an α-kinase domain. While its kinase domain regulates sensitivity to Mg^2+^ and cAMP, it is dispensable for the ion channel function. In glioblastoma, TRPM7 activates STAT3 signalling to upregulate ALDH1, thus driving CSC proliferation and invasion [33]. However, further investigation is needed to delineate the specific contributions of its kinase versus channel activities in modulating STAT3, ALDH1, and Notch pathways. Moreover, TRPM7 has been reported to be critical for neuroblastoma cell migration and SNAI2-driven metastasis [34]. In head and neck squamous cell carcinoma (HNSCC), high TRPM7 expression correlates with invasiveness, cisplatin resistance, and poor prognosis. Silencing TRPM7 not only impairs tumorsphere formation but also increases chemosensitivity [35].

Tumour-initiating cells (TICs), which include CSCs, exhibit elevated calcium sensitivity compared to their differentiated counterparts, likely due to enriched expression of ion channels. In glioma-initiating cells (GICs), this sensitivity correlates with neural stem cell markers such as GRIA1, NES, and FABP7 and diminishes upon differentiation. These findings highlight calcium homeostasis as a potential vulnerability in immature, stem-like cancer cells [76].

### 3.3. VGCCs and Their Functions

VGCCs, composed of α, β, δ, and γ subunits, regulate calcium entry and play critical roles in cellular functions [36]. Among them, CACNG4, a γ-subunit of VGCCs, has been implicated in several cancers [36]. Shiozaki et al. [37] investigated ion channel expression in gastric cancer stem cells (GCSCs) and found elevated levels of VGCC genes, especially *CACNA2D1* and *CACNB4*, in CD44-high cells isolated from an MKN74 gastric cancer cell line [37]. These GCSCs were more resistant to cisplatin but showed increased sensitivity to VGCC inhibitors, amlodipine and verapamil, which reduced tumorsphere formation and tumour growth when combined with cisplatin [37]. They also explored ion channel expression in hepatocellular carcinoma (HCC) CSCs (HepG2 line), revealing upregulation of several channel-related genes, including *CACNG4* [36]. Lee et al. [38] reported that ovarian cancer’s five-year survival rate remains low due to recurrence and drug resistance, largely driven by CSCs [38]. They investigated the effects of trimebutine maleate (TM) on ovarian CSCs using A2780-SP cells derived from A2780 ovarian cancer cells. They proved that TM showed selective toxicity to CSCs (GI_50_ ~0.4 µM) compared to non-CSCs (>100 µM), inducing cell cycle arrest and cell death. In mouse models, TM significantly reduced tumour growth [38]. TM targets overexpressed VGCCs and calcium-activated potassium channels (BKCa) channels in CSCs, and dual inhibition of these channels further reduced cell viability and stemness markers. Although they explained that TM effectively and selectively reduces stemness and proliferation in ovarian CSCs, potentially involving Wnt/β-catenin signalling [38], other pathways may also play a role. Additional signalling pathways should be investigated to fully clarify TM’s underlying mechanism of action.

### 3.4. Ca^2+^ Release Channels (CRCs)

CRCs like ryanodine receptors (RyRs) and inositol trisphosphate receptors (IP3Rs) regulate calcium flux from the endoplasmic reticulum and are increasingly linked to CSC functions [3]. RyR1 expression is elevated in chemotherapy-resistant BCSCs, where it promotes calcium release via the glutathione S-transferase omega 1 (GSTO1) pathway; blocking this channel reduces CSC markers like Nanog and tumour volume in mouse models [3]. Similarly, downregulation of RyR3 by miR-367 in medulloblastoma enhances stemness traits, including Oct4 and CD133 expression [3]. IP3Rs, though better known for roles in oncogenesis and stem cell differentiation, have also been implicated in CSC maintenance; loss of Selenoprotein K (SELENOK) impairs IP3R function in melanoma, reducing intracellular calcium and stemness gene expression such as PROM1 [3]. These findings suggest that both RyRs and IP3Rs contribute to CSC maintenance, though isoform-specific effects require further study.

## 4. Mechanosensitive Channels and Their Functions

Recently, mechanosensitive channels have gained significant attention for their emerging role in regulating CSCs. Piezo1, a mechanosensitive, calcium-permeable ion channel, regulates focal adhesion dynamics in normal cells by localizing to adhesions in a force-dependent manner [42]. It promotes adhesion maturation and disassembly via localized calcium influx, likely through calpain activation, in an integrin-dependent manner. However, in cancer cells, Piezo1 fails to localize to adhesions and has little effect on adhesion turnover, suggesting a tumour-associated functional shift [42]. Piezo1 supports colon cancer stem cell (CCSC) stemness via calcium/NFAT1 signalling [41]. Piezo1 knockdown reduced Yoda1-induced Ca^2+^ influx and nuclear NFAT1 protein, without affecting its mRNA. It also destabilized NFAT1 and suppressed stem-like traits, while Piezo1 overexpression increased nuclear NFAT1 and stemness. NFAT1 downregulation blocked Piezo1-driven sphere formation and gene expression [41]. In AML, PIEZO1 is upregulated and associated with better survival [40]. While activation has minimal effect, silencing PIEZO1 impairs proliferation, induces cell cycle arrest, and promotes apoptosis by disrupting DNA repair pathways [28]. In glioblastoma, exosomal circZNF800 from glioma stem-like cells promotes tumorigenesis by activating the PIEZO1/Akt pathway via miR-139-5p sponging. Silencing circZNF800 reduces GBM growth and improves survival in vivo. Exosomes from glioma stem-like cells (GSLCs) contribute to glioblastoma (GBM) aggressiveness by transferring specific circular RNAs (circRNAs) [39]. Zhang et al. [39] identified circZNF800 as highly enriched in GSLC-derived exosomes and associated with poor prognosis in GBM patients. CircZNF800 enhances GBM cell proliferation, migration, and survival by sponging miR-139-5p, thereby activating the PIEZO1/Akt signalling pathway. Silencing circZNF800 reduced tumour growth and improved survival in a GBM mouse model [39]. PIEZO1 links cell morphology and cancer progression. In breast cancer, PIEZO1 activation via YODA1 triggers epithelial–mesenchymal plasticity (EMP), a key step in metastasis and therapy resistance, by remodelling calcium signalling in response to shape changes [43].

## 5. Chloride Channels and Their Functions

Chloride ions, beyond their classical functions in maintaining membrane potential, cell volume, and intracellular pH, are increasingly recognized for their crucial roles in cancer biology, particularly in tumour cell proliferation, migration, and metastasis [44]. Chloride channels including calcium-activated chloride channels (CaCCs), voltage-gated chloride channels (ClCs), acid-sensitive chloride channels, and intracellular chloride channels (CLICs) are deeply involved in tumorigenesis, particularly through their support of CSC proliferation, survival, invasion, and adaptation to the tumour microenvironment [44,45,46,47,48,49,50].

CaCCs such as TMEM16A (also known as *ANO1*) are overexpressed in various cancers, including lung, breast, colorectal, and gliomas. ANO1 promotes tumour growth through activation of EGFR, PI3K/AKT, and MAPK signalling pathways. In colorectal cancer (CRC), its high expression is associated with poor prognosis, and pharmacological inhibition of ANO1 suppresses tumour growth by downregulating Wnt/β-catenin signalling [44,45]. Another CaCC, Bestrophin-1, has also been implicated in cancer progression, particularly in CRC and oral squamous carcinoma, potentially through modulation of calcium signalling and functional interaction with ANO1 [44].

ClCs such as ClC-2 and ClC-3 are upregulated in gliomas and contribute to enhanced proliferation and motility. Functional assays using antisense oligonucleotides and patch-clamp studies have demonstrated their role in chloride-mediated cell volume regulation and invasive behaviour within the dense extracellular matrix of the brain [46]. ClC-3 is also overexpressed in hepatocellular carcinoma (HCC), CRC, and nasopharyngeal and breast cancers, where it facilitates tumour progression via Wnt/β-catenin and IGF/ERK signalling. Silencing ClC-3 leads to reduced proliferation, suppressed IGF-1 signalling, and decreased tumour growth in breast cancer models [44]. ClC-4, another voltage-gated channel, supports CRC cell invasion and migration by maintaining pH homeostasis in acidic tumour microenvironments [44].

Acid-sensitive chloride channels, particularly *TMEM206*, are upregulated in CRC, breast, and liver cancers. TMEM206 enables tumour cells to adapt to extracellular acidosis and promotes CRC cell growth and invasion through the AKT/ERK pathway [44].

CLICs, mainly CLIC1 and CLIC4, are significantly overexpressed in several malignancies, including glioblastoma, prostate, colon, and HCC [44,47,49]. In glioblastoma, which is driven by CSCs, CLIC1 has been shown to be selectively active in CSCs but not in mesenchymal stem cells (MSCs) and is crucial for their proliferation and self-renewal [11]. It localizes to the plasma membrane during the cell cycle, where it mediates chloride flux in response to oxidative stress and pH shifts. Blocking CLIC1 disrupts these signals, effectively halting CSC growth without impacting normal stem cells [11]. Additionally, CLIC1 expression in glioblastoma stem cells (GSCs) correlates with poor prognosis, and its inhibition via RNA silencing or antibody targeting reduces proliferation, clonogenicity, and tumorigenicity both in vitro and in vivo [48,49,50].

## 6. Potassium (K^+^) Channels and Their Functions

Potassium (K^+^) channels are crucial regulators of membrane potential and cell signalling, classified into voltage-gated (Kv), inwardly rectifying (Kir), and two-pore (K2P) families [51]. In CSCs, specific K^+^ channels such as Kv1.3, KCa3.1, and voltage-sensitive human ERG (hERG, Kv11.1) regulate stemness, survival, and proliferation. Inhibition of these channels induces cell cycle arrest and reduces CSC proliferation, as observed in leukemia models [11,52]. Similarly, K^+^ channel tetramerization domain containing 12 (KCTD12), a K^+^ channel-associated protein, serves as a prognostic marker in gastrointestinal cancer. In colorectal CSC-like cells, its expression is reduced [11]. Silencing KCTD12 boosts self-renewal and drug resistance, while its overexpression suppresses these traits, highlighting its role in regulating CSC functions and chemoresistance. [11].

Various K^+^ channels including Kv, KCa, hEag, KATP, and K2P are overexpressed in CSCs from prostate, colon, lung, and breast cancers [51]. Blocking these channels suppresses tumour growth or enhances chemotherapy response. Kv (e.g., Eag1, HERG, Kv1.3), Kir3.1, and KCa (KCa1.1, KCa3.1) are especially linked to tumour progression [51]. In glioblastoma CSCs, KCa3.1 is upregulated and exhibits stronger currents in CD133^+^ cells [52].

In prostate cancer, overexpression of IKCa1 (KCa3.1) supports proliferation. Its activity hyperpolarizes the membrane and enhances calcium influx via TRPV6, potentially driving tumour growth [53]. In glioblastoma, M2 muscarinic receptor (M2 mAChR) activation suppresses CSC proliferation and migration partly by inhibiting IKCa currents. Direct IKCa inhibition with TRAM-34 reduces CSC motility and invadopodia formation [54]. In pancreatic CSCs, Shiozaki et al. [55] found upregulation of Kv channel genes in ALDH1A1^+^ cells from the PK59 line. Treatment with 4-aminopyridine (4-AP) selectively impaired CSC viability and reduced tumour growth in vivo. In breast cancer, multiple K^+^ channels are linked to CSC maintenance and tumour progression [56]. Kv1.3 is silenced in aggressive tumours via promoter methylation and associated with poor prognosis. Kv10.1 expression is elevated in invasive breast cancers, particularly in triple-negative breast cancer (TNBC), and these cells show sensitivity to astemizole. Kv11.1 (hERG1) is linked to better survival in ER-negative patients, whereas Kir3.1 correlates with metastasis in ER-positive tumours. KCNK9 (K2P) promotes migration in TNBC, while KCa1.1 and KCa3.1 are associated with higher tumour grade and metastasis [56].

## 7. Sodium (Na^+^) Channels and Their Functions

Na^+^ channels, particularly voltage-gated sodium channels (VGSCs), are membrane proteins that regulate Na^+^ influx and initiate action potentials [57]. In cancer, aberrant VGSC expression is increasingly linked to enhanced invasion, metastasis, and maintenance of CSC traits [57]. Although compounds like eicosapentaenoic acid and tamoxifen show anti-tumour effects by targeting VGSCs, their clinical use is limited due to poor subtype selectivity. However, recent efforts are focused on developing VGSC subtype-specific small molecules and peptides as promising therapeutic tools [57].

In glioblastoma stem cells (GSCs), Giammello et al. [58] reported that VGSCs help maintain the depolarized resting membrane potential (RMP), particularly during the G0 phase, supporting stemness. Blocking VGSCs induced GSC differentiation, reduced self-renewal, increased temozolomide sensitivity, and reactivated ERK signalling, suggesting a new approach for glioblastoma therapy [58]. TNBC, characterized by high plasticity and drug resistance, exhibits elevated activity of VGSCs [59]. Nav1.5 (SCN5A) enhances tumour cell invasion and drives epithelial-to-mesenchymal transition (EMT) [59]. Targeting VGSCs in TNBC has been linked to reduced stemness and tumour aggression [59].

## 8. ASICs and Their Functions

ASICs are voltage-insensitive sodium channels that detect extracellular acidity or proton levels [60]. Several ASIC subtypes, including ASIC1, ASIC1a, and ASIC3, have recently been identified in CSCs [60,61,62]. In glioblastoma stem cells (GSCs), which are key contributors to therapy resistance in malignant glioma, functional ASIC1a and ASIC3 channels are expressed [60]. In contrast, ASIC2, known for its anti-oncogenic role, is absent. Electrophysiological studies confirmed that ACCN2 and ACCN3 transcripts are translated into active ASIC1a- and ASIC3-containing channels. ASIC1b was present at low levels and largely non-functional [60]. Clusmann et al. [61] found that under prolonged acidosis, ASIC1a activation induces necrotic cell death, partially through necroptosis-like pathways. They proved that inhibiting ASIC1a or knocking it out protects GSCs from acid-induced death, while activating it reduces tumorsphere formation even at neutral pH [61]. Although their findings suggest that targeting ASIC1a could be a novel therapeutic approach by exploiting the acidic tumour microenvironment to eliminate CSC, no clear underlying mechanism has been described [61].

## 9. AQPs or Water Channels and Their Functions

AQPs, known primarily for their roles in water and solute transport, are now recognized for their broader involvement in key CSC-related processes and their regulations [63,64,65,66,67,68,69,70,71,72]. Recent research has described the crucial role of aquaporin channels in cancer, particularly in CSCs. Several isoforms, including AQP1, AQP3, AQP4, AQP5, AQP8 and AQP9, are expressed in CSC populations [63,64,65,66,67,68,69,70,71,72]. AQP1 overexpression in rat C6 glioma CSCs enhanced cell viability and migration, potentially promoting glioma progression through transcriptional networks involving Foxo4, Maz, and E2F factors [63]. AQP3 has emerged as a key oncogenic factor across multiple cancers [64,65,66,67]. In breast cancer, AQP3 is upregulated in ER-positive invasive ductal carcinoma, where it enhances migration, invasion, and EMT. Its expression is estrogen-inducible via an estrogen response element (ERE) in the promoter. Clinically, elevated AQP3 correlates with poor differentiation, lymph node metastasis, and premenopausal status [64]. Similarly, AQP3 is highly expressed in hepatocellular carcinoma (HCC), where it correlates with advanced stage, metastasis, and poor prognosis [65]. Mechanistically, AQP3 promotes stemness by enhancing CD133 expression through activation of the JAK/STAT3 pathway, which facilitates STAT3 phosphorylation, nuclear translocation, and histone H3 acetylation at the CD133 promoter [65,66]. Additionally, Liu et al. [67] reported that AQP3 inhibits differentiation and apoptosis in liver cancer stem cells (LCSCs) by suppressing components of the Wnt/GSK-3β/β-catenin pathway, including β-catenin, GSK-3β, and STAT3. Collectively, these findings position AQP3 as a key driver of cancer progression and stemness through distinct signalling mechanisms [64,65,66,67]. In glioblastoma (GBM), AQP4-loaded extracellular vesicles (EVs) influence tumour behaviour [68]. EVs containing AQP4 tetramers promote cell migration, while those with AQP4 orthogonal arrays of particles (OAPs) induce apoptosis, highlighting a novel mechanism by which AQP4 modulates the tumour microenvironment [68]. AQP5 is highly overexpressed in breast cancer, but not in normal tissue [69]. Its expression is linked to loss of polarity, increased proliferation, and poor prognosis, particularly in ER/PR-negative and HER2-positive cases. Gene amplification may drive this overexpression [70]. In gastric cancer, AQP5 serves as a surface marker of cancer stem cells (GC-CSCs), co-localizing with LGR5 [70]. It enhances growth, invasion, and tumour formation by activating autophagy and promoting ULK1 ubiquitination [70]. AQP8 is overexpressed in cervical cancer and promotes viability, migration, and EMT, while suppressing apoptosis [71]. It has been reported that AQP9 is expressed and significantly downregulated in LCSCs, and its restoration suppresses stemness by promoting reactive oxygen species (ROS) accumulation. This leads to reduced β-catenin–TCF4 interaction and enhanced β-catenin–FOXO3a association, ultimately inhibiting LCSC self-renewal [72].

### Intracellular Organelles Ion Channels and Their Functions

Intracellular organelle ion channels play essential roles in the survival and function of CSCs by regulating ion homeostasis, apoptosis, and metabolic signalling. Mitochondrial ion channels such as the mitochondrial calcium uniporter (MCU) are critical for tumour growth, influencing cell cycle progression and energy metabolism essential for CSC maintenance [77]. MCU-driven mitochondrial calcium uptake enhances breast cancer cell migration via store-operated Ca^2+^ entry; inhibition of MCU suppresses metastasis in vivo and slows tumour growth through impaired mitochondrial redox signalling [78]. Additionally, ER-resident channels like TMCO1 modulate calcium dynamics and resistance to apoptosis, contributing to CSC survival under therapeutic stress [79]. Synthetic ion transporters targeting mitochondrial and lysosomal membranes have shown potential in selectively inducing apoptosis in CSCs, revealing novel avenues for anti-CSC therapies [80]. Recently, Zheng et al. [81] proved that circular RNA hsa_circ_0007905 is upregulated in cervical cancer (CC) and enriched in cancer stem-like cells [81]. Its knockdown impairs proliferation, invasion, self-renewal, and promotes apoptosis. Mechanistically, it sponges miR-330-5p, relieving repression of VDAC1. Restoring VDAC1 or inhibiting miR-330-5p reverses these effects. They suggested that this axis may serve as a therapeutic target in CC [81]. These findings highlight the functional significance of organellar ion channels in CSC biology and emphasize their therapeutic potential in targeting resistant cancer populations.

**Figure 2:**
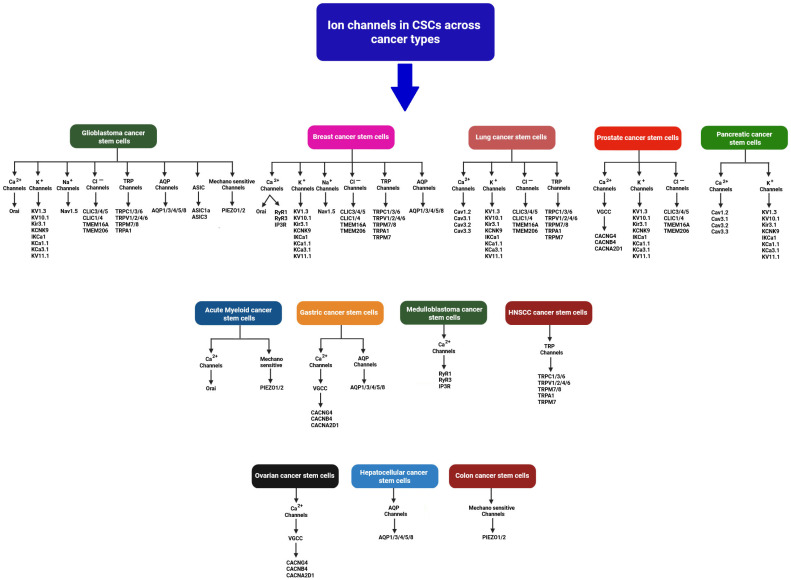
Ion channels in cancer stem cells (CSCs) across various cancer types.

To enhance clarity and comprehension, all the ion channels discussed in Section 2, Section 3, Section 4, Section 5, Section 6, Section 7, Section 8 and Section 9 have been systematically summarized based on their specific expression across diverse cancer stem cell types, as schematically illustrated in Figure 2.

This schematic depicts the distribution of ion channels identified in cancer stem cells (CSCs) across a broad spectrum of solid and hematological malignancies, including glioblastoma, breast, lung, prostate, pancreatic, ovarian, gastric, hepatic, colorectal, medulloblastoma, head and neck squamous cell carcinoma (HNSCC), and acute myeloid leukemia [19,25,39,52,61,71]. Ion channels are categorized based on ion selectivity (e.g., Ca^2+^, K^+^, Na^+^, Cl^−^), family classification (e.g., TRP, AQP, VGCC), or functional type (e.g., mechanosensitive). The schematic highlights both overlapping and cancer-type-specific expression patterns, underscoring the critical role of ion channels in CSC maintenance and signalling [27,33,56,65]. These insights offer potential avenues for the development of CSC-targeted therapies. All channel types and their functions are described in detail in the main text.

## 10. Interplay Between Ion Channels and Epigenetic Control in CSCs

Understanding ion channel expression and function in CSCs reveals their role in regulating key cellular processes. Epigenetic mechanisms further modulate these channels, affecting their expression, localization, and activity. The next section explores how this interplay supports CSC maintenance, plasticity, and therapy resistance.

CSCs, recognized as subpopulations within tumours, contribute significantly to tumour heterogeneity through both genetic and epigenetic mechanisms [82]. This heterogeneity enhances the adaptability of cancer cells, facilitating invasion and resistance to therapy [83]. Epigenetic regulation—comprising DNA methylation, histone modifications, and non-coding RNAs—maintains CSC plasticity and stemness without altering the DNA sequence [84,85]. Emerging evidence reveals a dynamic crosstalk between ion channel signalling and epigenetic machinery in CSCs [86]. Ion channels, especially those mediating calcium influx like the store-operated Ca^2+^ channel Orai1, activate intracellular cascades such as CaMKII-AKT/PI3K and MAPK pathways, which in turn upregulate EMT drivers like TWIST (TWIST related protein1 ir Twist1), thereby promoting CSC-like states [87,88]. TWIST is not only central to EMT but also facilitates tumour initiation and metastasis. Epigenetic mechanisms reciprocally modulate ion channel expression; for example, DNA methylation silences Kv1.3 in colorectal cancer, impacting cellular excitability and tumour behaviour [88]. Additionally, post-translational modifications of histones—such as methylation and acetylation—govern transcription of key epithelial markers like CDH1, often repressed by the polycomb protein EZH2 [89]. In BCSCs, hyperactivation of the nuclear respiratory factor 2 (NRF2) pathway is mediated by the epigenetic reader Zinc Finger MYND-Type Containing 8 (ZMYND8), which enhances antioxidative capacity and promotes resistance to oxidative damage and ferroptosis [90]. Emerging evidence suggests that this NRF2-ZMYND8 axis modulate the expression and activity of redox-sensitive ion channels, such as TRP channels and calcium-permeable channels, thereby contributing to CSC survival, metabolic flexibility, and therapeutic resistance under oxidative stress conditions [91]. TRPM2, a member of the TRP melastatin subfamily, is highly expressed in variety of tumours and CSCs and plays a key role in protecting cell viability by modulating oxidative stress [91,92]. Interestingly, the inhibition of TRPM2 channels results in mitochondrial dysfunction, elevated ROS, and reduced levels of antioxidant cofactors such as GSH, NADPH, and NADH—effects linked to suppressed NRF2 signalling and decreased expression of its antioxidant gene targets. Importantly, TRPM2-dependent stabilization of NRF2 involves IQGAP1 and calcium signalling, pointing toward a regulatory loop where epigenetic factors like ZMYND8 may indirectly influence TRPM2 expression and function, further coordinating redox balance and stemness in CSCs [91,92]. Recent research demonstrated that mitoepigenetic dysregulation in CSCs affects mitochondrial function and redox balance, contributing to tumour progression. Recent findings show that AIF-induced mitochondrial Ca^2+^ overload, due to impaired mitochondrial Ca^2+^ uniporter (MCU) regulation, activates calpain and retrograde signalling, enhancing oncogenic gene expression in CSCs. This ROS–Ca^2+^–calpain axis, integrated with epigenetic modulation of mitochondrial DNA, links mitochondrial dynamics to CSC survival and aggressiveness in numerous tumours [75,93]. In glioblastoma stem-like cells (GSCs), VDAC1 depletion has been shown to trigger extensive epigenetic reprogramming, leading to changes in chromatin organization and transcriptional activity. Specifically, silencing of VDAC1 in GSCs results in reduced mitochondrial metabolism and NAD^+^ levels, which in turn downregulates the activity of sirtuin deacetylases SIRT1 and SIRT6—key epigenetic regulators [94,95]. This inhibition leads to hyperacetylation of histone H3 (at Lys9 and Lys27) and histone H4, altering the expression of genes involved in stemness and oncogenic signalling. Notably, this also affects the expression of calcium and potassium ion channels such as CACNA1C (L-type calcium channel) and KCNMA1 (BK channel), both of which are epigenetically regulated and critical for CSC maintenance and invasiveness [95]. These bidirectional interactions between ion channels and epigenetic modifiers represent a complex regulatory axis, critical to CSC maintenance, tumour progression, and therapeutic resistance.

## 11. Ion Channel-Targeted Therapies in CSCs

**Figure 3:**
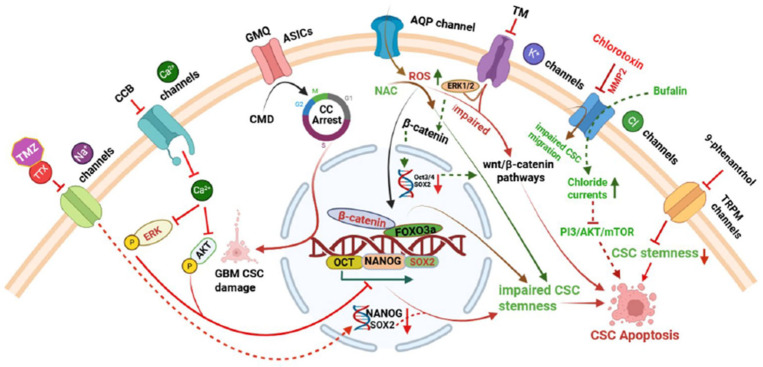
Targeting ion channels in cancer stem cells (CSCs) as a promising therapeutic strategy.

Targeting ion channels presents a promising therapeutic approach, especially in cancer, where their dysregulation contributes to tumour growth, invasion, and treatment resistance. By modulating these channels, it is possible to disrupt critical signalling pathways and impair CSC functions, contribution a novel avenue for more effective treatments [96]. CSC ion channel blockers have shown significant potential in cancer therapy, influencing tumour cell proliferation, differentiation, apoptosis, and metastasis [97]. This chapter explores the emerging strategies aimed at modulating ion channel activity to selectively target CSCs, offering novel avenues for more effective and durable cancer treatments (Table 2 and Figure 3).

Pharmacological modulation of ion channels interferes with cancer stem cell (CSC) stemness and promotes apoptosis through diverse mechanisms. Inhibition of voltage-gated Na^+^ channels by temozolomide (TMZ) and tetrodotoxin (TTX) reduces SOX2 and NANOG expression (indicated by dotted red lines), suppresses glioma stem cell (GSC) self-renewal, and induces G1-phase cell cycle arrest [61,110]. L-type Ca^2+^ channel blockers, including manidipine, lomerizine HCl, and benidipine HCl, attenuate stemness by suppressing AKT/ERK signalling and triggering apoptosis [99]. Activation of ASIC3 with GMQ causes selective depolarization of glioblastoma CSCs, resulting in cell damage [73,111]. In ovarian CSCs, trimebutine maleate (TM) inhibits voltage-gated and Ca^2+^-activated K^+^ channels, downregulates stemness markers, and suppresses Wnt/β-catenin signalling. Activation of aquaporin (AQP) channels enhances reactive oxygen species (ROS) accumulation, thereby impairing Wnt/β-catenin signalling and inducing apoptosis, whereas ROS scavenging by NAC restores β-catenin activity and CSC stemness [72]. Chloride channel modulators such as chlorotoxin and bufalin inhibit CSC migration and survival by targeting MMP-2 and the PI3K/AKT/mTOR pathway, respectively (indicated by green dotted lines and red dotted lines for apoptosis). In breast CSCs, TRPM4 inhibition using 9-phenanthrol diminishes stemness and facilitates apoptosis [33]. All mechanisms and therapeutic strategies are described in detail in the main text.

The therapeutic potential of targeting various calcium channels in CSCs is increasingly recognized for its ability to disrupt tumour progression and overcome treatment resistance. FDA-approved manidipine and lacidipine inhibit L- and T-type voltage-gated calcium channels, leading to reduced sphere formation, viability, and proliferation of ovarian CSCs [98]. They also induce apoptosis and suppress stemness by inhibiting the AKT and ERK signalling pathways, which are crucial for maintaining CSC properties [98]. Mibefradil inhibits the T-type calcium channel Cav3.2, which is overexpressed in glioblastoma stem-like cells (GSCs) [99]. This inhibition leads to decreased proliferation, survival, and stemness of GSCs, and sensitizes them to temozolomide chemotherapy. This effect is mediated through suppression of the pro-survival AKT/mTOR pathway and activation of apoptotic regulators such as survivin and BAX [99]. SOCE channels are essential for calcium influx in glioblastoma CSCs [26]. Pharmacological inhibition of SOCE reduces proliferation, impairs self-renewal, and decreases the expression of stem cell markers like SOX2, thereby targeting the CSC population responsible for tumour recurrence [26]. Verapamil, a Ca^2+^ channel blocker, targets MDR-related proteins, reducing proliferation and inducing apoptosis in gemcitabine-resistant pancreatic CSCs [100]. In GBM, combining the KCa3.1 inhibitor TRAM-34 with temozolomide (TMZ) significantly decreases DNA synthesis and CSC survival compared to TMZ alone, also reducing tumour cell infiltration [101,102].

TRPC6 supports breast cancer stem cell traits and chemoresistance by regulating integrin α6 mRNA splicing [103]. It suppresses epithelial splicing regulatory protein 1 (ESRP1), promoting the α6B splice variant, which activates the transcriptional co-activator with pDZ-binding motif (TAZ) and reduces Myc expression. Blocking TRPC6 disrupts this pathway, sensitizing TNBC cells to chemotherapy [103]. Compounds like waixenicin A, derived from *S. edmondsoni 14*, irreversibly inhibit TRPM7 channels but lack specificity, limiting their therapeutic use [33]. Activation of STAT3 and Notch pathways regulates ALDH1 genes in glioma stem cells (GSCs), suggesting promising targets for glioma therapy [33].

In hepatocellular carcinoma CSCs, the VGCC subunit CACNG4 is implicated in maintaining stemness, and its inhibition by amlodipine suppresses CSC characteristics [36].

Potassium (K^+^) channel inhibition promotes apoptosis and may reverse multidrug resistance (MDR). Kv1.3 inhibitors like margatoxin (MgTX) and non-selective blockers such as 4-aminopyridine (4-AP) suppress metastasis in prostate cancer and reduce lung cancer cell growth, with MgTX also promoting apoptosis by inducing G1-S phase arrest [104].

Glioblastoma CSCs show strong resistance to BCNU (bis-chloroethylnitrosourea) chemotherapy; however, combining BCNU with the chloride channel inhibitor DIDS (4,4′-diisothiocyanostilbene-2,2′-disulfonic acid) promotes apoptosis and reduces proliferation [105]. CLIC1 contributes to this resistance and may act as a drug efflux channel. Inhibiting CLIC1, either pharmacologically (e.g., with metformin) or via RNA interference, enhances CSC sensitivity to treatment. Similarly, blocking the CLCN3 channel also suppresses CSC proliferation. These findings highlight chloride and calcium channel inhibitors as potential tools to overcome multidrug resistance in cancer therapy [105]. New biguanide compounds Q48 and Q54 block CLIC1 more effectively than metformin, reducing GSC proliferation, invasion, and self-renewal with minimal toxicity, showing promise as targeted glioblastoma therapies [49].

Recent studies have highlighted the therapeutic potential of targeting ASICs in CSCs, particularly within glioblastoma, where acidic tumour microenvironments activate these channels. ASIC1a has been shown to induce necroptosis in GSCs through a RIPK1-dependent pathway [60]. This cell death mechanism can be inhibited by the ASIC1a antagonist PcTx1, indicating that pharmacological modulation of ASIC1a could be a viable therapeutic strategy [60]. Additionally, ASIC3 has been identified as a contributor to GSC proliferation and migration, with its inhibition leading to reduced tumour growth, suggesting that ASIC3 is another promising target for therapy [73]. Beyond glioblastoma, ASIC1a’s role extends to other cancers; for instance, in breast and prostate cancers, its activation leads to reactive oxygen species (ROS) production and activation of survival pathways like AKT and NF-κB, while in pancreatic cancer, it promotes epithelial–mesenchymal transition via RhoA signalling [106]. These findings describe the significance of ASICs in cancer progression and the potential of ASIC-targeted therapies across various tumour types.

AQP channels also regulate CSC functions, offering novel targets for cancer therapy. In gastric cancer, AQP3 is overexpressed and correlates with the CSC marker CD44. This overexpression enhances stem-like traits in gastric cancer cells by activating the Wnt/GSK-3β/β-catenin signalling pathway. Experimental manipulation of AQP3 levels directly influences tumorigenic potential and self-renewal capacities, emphasizing its role in maintaining CSC characteristics. Targeting AQP3 could, therefore, disrupt these pathways and diminish CSC-driven tumour growth [107]. Similarly, AQP5 is upregulated in gastric cancer stem cells and contributes to their self-renewal by modulating autophagy. Inhibiting AQP5 expression has been shown to reduce the stemness of these cells, suggesting that AQP5 is integral to the maintenance of CSC properties and represents a potential therapeutic target [108]. In hepatocellular carcinoma, AQP9 expression is typically reduced in liver CSCs. Restoring AQP9 levels suppresses CSC characteristics by increasing reactive oxygen species (ROS) accumulation, which in turn inhibits β-catenin activity and promotes its interaction with FOXO3a. This mechanism leads to the attenuation of stemness features in liver CSCs, highlighting AQP9 as a promising target for therapeutic intervention [80]. Collectively, these findings emphasize the diverse roles of AQPs in sustaining CSC properties across different cancer types. Therapeutic strategies aimed at modulating AQP function or expression could, therefore, be effective in targeting CSCs and impeding cancer progression.

Many ion channels enriched in GSCs belong to broader families with overlapping functions, making it challenging to target specific channels therapeutically [109]. To address this, Pollak et al. [109] analyzed enrichment patterns at the family level, identifying 12 ion channel families (including epithelial Na^+^ channels, GABA_A receptors, and ionotropic glutamate receptors) with significant GSC-specific expression. Pharmacological blockade of these enriched ion channels, such as with TTX, TEA, 4-AP, CPP, CNQX, ω-Conotoxin MVIIC, and CdCl_2_, significantly reduced GSC viability in vitro, confirming their functional relevance and therapeutic potential [109].

Taken together, the expanding landscape of ion channel-targeted therapies offers a promising avenue for eradicating CSCs and overcoming treatment resistance. However, to fully realize their clinical potential, these strategies must be integrated with precise diagnostic tools, molecular profiling, and advanced drug delivery technologies.

## 12. Challenges and Strategies in Ion Channel Research for CSCs

Ion channels are key regulators of CSC functions, but despite their therapeutic potential, significant challenges persist in the research and development of ion channel-targeted drugs for CSCs [96,112]. One of the primary obstacles is the remarkable heterogeneity and plasticity of CSCs [96,113,114,115]. CSCs display intra- and inter-tumoral heterogeneity, and their phenotypic plasticity enables dynamic shifts between stem-like and differentiated states, often accompanied by changes in ion channel expression and function [96,113,114,115]. This variability complicates the development of universal ion channel-targeted strategies [96,113,114,115]. Moreover, ion channels in CSCs frequently exhibit functional redundancy. For instance, upregulated expression of several voltage-gated potassium (Kv) channels, including KCNB1, KCNC1, and KCND1, has been reported in pancreatic CSCs (PK59 CSCs) [116]. The inhibition of KCNB1 using 4-aminopyridine (4-AP) reduced CSC proliferation, suggesting that despite the overexpression of multiple Kv channels, targeting specific ones like KCNB1 can still impact CSC viability. This underscores potential redundancy among Kv channels in supporting CSC functions [116].

Another key challenge is safety. Many ion channels essential for CSC maintenance are also expressed in normal stem cells and in vital organs such as the heart and brain, raising concerns about off-target effects [96]. To address this, the development of CSC-specific delivery systems or modulators with high selectivity is essential. Further complicating this issue is the inadequacy of conventional cancer models in mimicking the CSC niche and ion channel expression patterns [117]. Sphere-forming assays and xenograft models, while useful, are limited in both throughput and physiological relevance. Additionally, assessing ion channel function in rare, non-adherent CSC populations using traditional electrophysiological tools is technically difficult. Although high-throughput, label-free technologies suitable for CSCs are emerging, they remain in developmental stages [118]. The CSC microenvironment-characterized by hypoxia, acidosis, and interactions with stromal components—further modulates ion channel activity [96,119]. Hypoxia, for example, can alter both expression and gating properties of ion channels, thereby affecting drug sensitivity. Thus, ion channel-targeted therapies must be evaluated in models that reflect the true CSC microenvironment to ensure clinical translatability. Even the identification of CSCs remains technically challenging due to overlapping molecular markers with normal stem cells. However, promising therapeutic targets like CD47 have shown success in reducing CSC populations using anti-CD47 antibodies, especially in AML [96,120].

To overcome the challenges associated with targeting ion channels in CSCs, a multidisciplinary, precision medicine-oriented approach is essential. Several therapeutic strategies are currently under investigation, which can be broadly categorized based on their translational maturity. Organizing these approaches according to their stage of development—notably preclinical discovery, early-phase clinical evaluation, and late-stage clinical trials or approved use—provides strategic clarity. This framework not only facilitates prioritization of candidates for experimental validation and clinical translation but also helps in aligning efforts across research and regulatory domains. Some strategies have demonstrated feasibility in preclinical or early clinical settings. These include the use of ion channel inhibitors such as 4-AP targeting KCNB1 in pancreatic CSCs [116], CD47-targeted therapies like anti-CD47 antibodies in AML [96,120], nanoparticle-based drug delivery systems that selectively transport ion channel modulators to CSC niches [12], and targeting ion channel-regulated signalling pathways such as Ca^2+^-dependent Wnt/β-catenin, Notch, and NFAT, especially when combined with conventional therapies [18]. These approaches show promise for near-term translation into clinical applications.

On the other hand, several strategies remain largely exploratory or theoretical. These include the development of CSC-specific ion channel biomarkers using single-cell transcriptomics and spatial proteomics [112,121,122], though their integration into CSC-focused applications is still evolving [123]. Combining functional electrophysiological profiling with gene expression data is also considered important for tracking ion channel activity in situ and across various CSC states, including quiescence and EMT [124], but such integration remains limited in current CSC research. Similarly, patient-derived organoids and 3D tumour models offer greater physiological relevance for assessing drug efficacy and safety [125], yet their use in high-throughput ion channel screening remains under development. Structure-based drug design using cryo-EM and computational modelling holds great potential for generating selective inhibitors that target CSC-specific ion channel conformations [126]. While cryo-EM has advanced the structural understanding of many ion channels [127], its translation to CSC-targeted drug design is still conceptual. Another emerging idea is mitochondrial ion channel targeting NIR II-responsive nanoparticles, which offers a novel mechanistic route but currently lacks CSC-specific validation [128].

In summary, while some ion channel-targeted approaches are nearing translational application, many others remain in developmental or conceptual stages. A clear, stage-wise roadmap that distinguishes between mature, actionable strategies and those requiring further validation is essential. Such organization will help prioritize research directions and reveal the full therapeutic potential of ion channel modulation in eliminating CSCs and improving long-term cancer outcomes.

## 13. Conclusions and Future Perspectives

Although substantial advancements in cancer therapies have extended patient survival, drug resistance—largely driven by CSCs—remains a significant clinical hurdle. A growing body of evidence highlights the pivotal role of ion channels in CSC biology, including their contributions to tumour initiation, progression, and resistance to therapy, hence earning the designation of “oncogenic channels” [115]. Aberrant expression and dysregulation of these channels enable CSCs to evade conventional treatments through diverse mechanisms. Targeting ion channels in CSCs approaches a promising strategy to overcome therapeutic resistance. Ideal ion channel targets should exhibit minimal expression in normal tissues, high expression in tumour tissues, selective ligand binding, and low toxicity profiles [18,115]. However, few candidates have progressed into early-phase clinical trials, indicating the pressing need for focused translational research. Existing ion channel inhibitors—already approved for cardiac or neurological disorders—are now being repurposed in oncology, showing encouraging preclinical and early clinical results [17]. Emerging findings emphasize that CSC-specific ion channels, such as Na^+^, K^+^, Ca^2+^, and Cl^−^ channels, modulate key processes including proliferation, invasion, and apoptosis. For instance, inhibition of IKCa and BKCa channels has shown therapeutic promise in glioblastoma models [115], where glioma stem cells exhibit high malignancy and resistance. These results emphasize the translational value of targeting CSC-associated ion channels, particularly in highly aggressive and heterogeneous cancers such as glioblastoma, where standard therapy still relies on maximal resection, radiotherapy, and chemotherapy [129]. Therefore, understanding of ion channel expression and regulation in CSCs is crucial. Herein, we explore the diverse range of ion channels present in CSCs, highlighting recent insights into their roles in regulating CSC behaviour, functional characteristics, and cellular dynamics. Particular attention is given to ASICs and AQPs, as their crucial involvement in controlling CSC behaviour and dynamics has remained underexplored in prior reviews. ASIC1 and ASIC3 promote CSC survival and invasiveness under acidic tumour microenvironments by activating PI3K/Akt and ERK/MAPK pathways [106]. Inhibiting these channels has reduced tumour growth in preclinical models, making them compelling pH-responsive therapeutic targets. Similarly, overexpression of AQP1, AQP3, AQP4, AQP5, and AQP8 has been reported in CSC-enriched tumours, where they regulate cell motility, redox signalling, and chemoresistance [64,65,66,67,68,69,70,71,72]. Among them, AQP3 is particularly implicated in promoting EMT [64]. Small-molecule inhibitors and siRNAs targeting AQPs have demonstrated success in sensitizing tumours to standard therapies [17]. Moreover, advances in drug delivery and natural product pharmacology have shown promise. Natural compound-based modulators like limonin and silibinin, which inhibit TMEM16A, exhibit anticancer potential in lung and prostate cancers [115]. Additionally, existing ion channel blockers such as verapamil, mibefradil, and riluzole have shown clinical benefit when repurposed for cancers like metastatic breast cancer, glioblastoma, and melanoma brain metastases, respectively [115,121]. Despite these advances, a major bottleneck remains: the absence of robust biomarkers to predict therapeutic response to ion channel modulators. Therefore, future studies should emphasize the discovery and validation of such biomarkers through genomics, proteomics, and metabolomics. Progress in biomarker development, rational drug design, and precision delivery systems will be crucial to realizing the full potential of ion channel-targeted therapies [115,130].

Intracellular ion channels such as VDACs in the outer mitochondrial membrane and the MCU in the inner mitochondrial membrane have emerged as key regulators of CSC maintenance. However, their potential as therapeutic targets remain largely unexplored. Similarly, ER-resident Ca^2+^ channels like the ryanodine receptor (RyR) and IP3 receptor play crucial roles in cancer stemness, yet their modulation for CSC-targeted therapy has not been extensively investigated. Future research focusing on these ion channels may reveal novel strategies for cancer treatment. Exploring these underexplored areas holds promise for advancing targeted therapies against CSCs [3,94].

While CSCs have been identified in a wide range of tumour types, comprehensive data on ion channel expression within these cells remain limited. Further research is essential to systematically map ion channel profiles across diverse CSC populations. In this review, we have proposed a strategic roadmap that prioritizes the translational potential of targeting ion channels in CSCs, with emphasis on the following key points: (i) Development of ion channel-based biomarkers for CSC identification and therapeutic targeting: Ion channels such as Kv11.1 (hERG1) and TRPM7 have been shown to be overexpressed in CSC-like populations across several cancers, including breast and colorectal cancers [131,132]. Research also demonstrated that hERG1 expression is enriched in colon CSCs and its inhibition reduces tumour sphere formation, suggesting its utility as both a biomarker and therapeutic target [131]. These channels not only contribute to self-renewal and invasiveness but are also associated with poor prognosis, making them promising biomarkers. Additionally, TMEM16A (ANO1) has emerged as a potential CSC marker in head and neck squamous cell carcinoma [133]. (ii) Integration of single-cell omics to resolve ion channel heterogeneity in CSCs: Advancements in single-cell RNA sequencing (scRNA-seq) enable detailed characterization of ion channel expression at the individual cell level, helping to map CSC heterogeneity more accurately. This approach can reveal rare CSC subpopulations and their dynamic ion channel profiles under therapeutic pressure [134]. A recent single-cell transcriptomic study in glioblastoma identified specific potassium channel isoforms enriched in therapy-resistant CSCs, highlighting their role in survival and adaptation [135]. Recently developed technologies like CITE-seq and Patch-seq combine electrophysiological and transcriptomic profiling, offering powerful tools for functionally characterizing ion channel activity in rare CSCs [136]. (iii) AI-driven profiling and machine learning for patient stratification: Artificial intelligence and machine learning models are increasingly used to analyze large-scale ion channel expression datasets and correlate them with patient outcomes. AI tools can stratify patients based on CSC-associated ion channel signatures and predict responsiveness to ion channel modulators. Using deep learning, researchers have identified ion channel gene expression signatures predictive of drug resistance and survival in lung adenocarcinoma [137]. These strategies could inform targeted therapy decisions in patients with CSC-rich tumours. AI-based classifiers trained on TCGA datasets have successfully used ion channel-related genes for prognostic modelling in hepatocellular carcinoma [138], demonstrating their utility in precision oncology. In the future, addressing the potential of AI models to predict CSC behaviour or ion channel dysregulation over time would be both highly relevant and impactful. Moreover, applying AI approaches to study ion channel dysregulation and CSC formation and maintenance or to evaluate the therapeutic potential of specific ion channel inhibitors would be both appropriate and valuable.

## Figures and Tables

**Table 1:** Ion channel expression and functions in cancer stem cells (CSCs).

Ion Channel Type	Specific Channels/Subtypes	Cancer Type/Model System	Transported Ion(s)	Functional Role in CSCs	Reference(s)
SOCE Channels	Orai channels	Breast (HER2+, TNBC), AML, OSCC, Glioblastoma	Ca^2+^	Promote self-renewal, drug resistance, glycolysis, proliferation	[8,19,20,21,22,23,24,25]
	SPCA2, SK3, Kv10.1	Breast Cancer	Ca^2+^, K^+^	Store-independent Ca^2+^ entry, stemness support	[8,19]
TRP Channels	TRPC1/3/6, TRPV1/2/4/6, TRPM7/8, TRPA1	Glioblastoma, TNBC, Breast, NSCLC, Bladder, HNSCC, Neuroblastoma	Ca^2+^, Na^+^, Mg^2+^	Control proliferation, chemoresistance, migration, metastasis	[26,27,28,29,30,31,32,33,34,35]
	TRPM7	Lung, GBM, HNSCC	Ca^2+^, Mg^2+^	Enhances stemness, EMT, invasion, survival	[28,29,33,34,35]
VGCCs	CACNG4, CACNA2D1, CACNB4	Gastric, HCC, Ovarian	Ca^2+^	Mediate proliferation, drug resistance, survival	[36,37,38]
	Cav1.2, Cav3.2, Cav3.1, Cav3.3	Prostate, Breast, NSCLC, Pancreatic	Ca^2+^	EMT, SNAIL, HIF-1α, RhoA, TGFβ, VEGF, PI3K/AKT/mTOR	[25,26,27,36,39]
Calcium Release Channels (CRCs)	RyR1, RyR3, IP3Rs	Breast, Medulloblastoma, Melanoma	Ca^2+^	ER Ca^2+^ release, CSC enrichment, stemness marker regulation (Nanog, Oct4, CD133, PROM1)	[3]
Mechanosensitive Channels	PIEZO1, PIEZO2	Colon, AML, GBM, Breast	Ca^2+^	Regulate stemness, adhesion dynamics, tumorigenesis, Ca^2+^ influx, EMT, immune evasion	[39,40,41,42,43]
Chloride Channels	TMEM16A, Bestrophin-1, ClC-2/3/4, TMEM206, CLIC1/4	Lung, Breast, CRC, Glioma, HCC, Prostate	Cl^−^	EGFR, MAPK, PI3K/AKT, Wnt/β-catenin, oxidative stress, pH regulation, proliferation	[11,44,45,46,47,48,49,50]
Potassium Channels	Kv1.3, Kv10.1, Kv11.1, Kir3.1, KCNK9, IKCa1, KCa1.1, KCa3.1	Prostate, Colon, Lung, Breast, Pancreatic, GBM	K^+^	Regulate membrane potential, proliferation, EMT, ERK, JAK/STAT3, hypoxia	[51,52,53,54,55,56]
Sodium Channels (VGSCs)	Nav1.5	GBM, TNBC	Na^+^	Membrane potential, ERK activation, c-Myc, temozolomide resistance	[57,58,59]
ASICs	ASIC1a, ASIC3	GBM	H^+^ (protons)	pH sensing, necroptosis, acidic tumour suppression	[60,61,62]
Aquaporins (AQPs)	AQP1, AQP3, AQP4, AQP5, AQP8	Glioma, Breast, HCC, Cervical, Gastric	H_2_O, Glycerol, H_2_O_2_	EMT, JAK/STAT3, CD133, autophagy, TRIM21, Wnt/β-catenin	[63,64,65,66,67,68,69,70,71,72]

Abbreviations: CSC—cancer stem cell; SOCE—store-operated calcium entry; AML—acute myeloid leukemia; OSCC—oral squamous cell carcinoma; HER2—human epidermal growth factor receptor 2; TNBC—triple-negative breast cancer; SPCA2—secretory pathway Ca^2+^ ATPase 2; SK3—small conductance calcium-activated potassium channel 3; TRP—transient receptor potential; TRPC—TRP canonical; TRPV—TRP vanilloid; TRPM—TRP melastatin; TRPA1—TRP ankyrin 1; VGCC—voltage-gated calcium channel; CACNG4—calcium voltage-gated channel auxiliary subunit gamma 4; CACNA2D1—calcium voltage-gated channel auxiliary subunit alpha2delta 1; CACNB4—calcium voltage-gated channel auxiliary subunit beta 4; Cav—voltage-gated calcium channel alpha subunit; EMT—epithelial-to-mesenchymal transition; SNAIL—zinc finger protein SNAI1; HIF-1α—hypoxia-inducible factor 1-alpha; RhoA—Ras homologue family member A; TGFβ—transforming growth factor beta; VEGF—vascular endothelial growth factor; PI3K/AKT/mTOR—phosphoinositide 3-kinase/protein kinase B/mammalian target of rapamycin; RyR—ryanodine receptor; IP3R—inositol 1;4;5-trisphosphate receptor; PROM1—prominin-1 (CD133); PIEZO—mechanosensitive ion channel PIEZO; ClC—chloride channel family; TMEM—transmembrane protein; CLIC—chloride intracellular channel; KCa—calcium-activated potassium channel; IKCa—intermediate conductance KCa channel; Kir—inward rectifier potassium channel; KCNK—potassium channel subfamily K; VGSC—voltage-gated sodium channel; Nav—sodium voltage-gated channel; ASIC—acid-sensing ion channel; AQP—aquaporin; HNSCC—head and neck squamous cell carcinoma; GBM—glioblastoma multiforme; CRC—colorectal cancer; HCC—hepatocellular carcinoma; ERK—extracellular signal-regulated kinase; JAK/STAT3—Janus kinase/signal transducer and activator of transcription 3; TRIM21—tripartite motif containing 21; ER—endoplasmic reticulum.

**Table 2:** Ion channel-targeted therapeutic approaches in cancer stem cells (CSCs).

Ion Channel Type	Channel Name/Subtype	Ion Transported	Inhibitor/Drug/Compound	CSC Type/Cancer	Effect on CSCs	Clinical Status	Reference
Voltage-Gated Ca^2+^ Channels (VGCCs)	L- and T-type (e.g., Cav1.2, Cav3.2)	Ca^2+^	Manidipine, Lacidipine	Ovarian CSCs	↓ Sphere formation, proliferation, stemness; apoptosis; inhibits AKT/ERK	FDA-approved (antihypertensives); preclinical for CSCs	[98]
Voltage-Gated Ca^2+^ Channel (T-type)	Cav3.2	Ca^2+^	Mibefradil	Glioblastoma stem-like cells (GSCs)	↓ Proliferation, survival, stemness; sensitizes to temozolomide; suppresses AKT/mTOR, activates BAX	Withdrawn FDA drug; preclinical in GBM CSCs	[99]
Store-Operated Ca^2+^ Entry (SOCE)	Orai1/STIM1	Ca^2+^	SOCE inhibitors	Glioblastoma CSCs	↓ Proliferation, self-renewal, SOX2 expression	Experimental compounds; preclinical	[26]
Ca^2+^ Channels (non-specific)	—	Ca^2+^	Verapamil	Pancreatic CSCs	Targets MDR proteins; ↓ proliferation; ↑ apoptosis in gemcitabine-resistant CSCs	FDA-approved (cardiac); preclinical in CSCs	[100]
Ca^2+^-Activated K^+^ Channel	KCa3.1	K^+^	TRAM-34 + Temozolomide	GBM CSCs	↓ DNA synthesis, CSC survival, tumour infiltration	TRAM-34 preclinical; Temozolomide FDA-approved	[101,102]
TRP Channel	TRPC6	Ca^2+^/Na^+^	TRPC6 inhibitors	Triple-negative breast CSCs	Disrupts integrin α6 splicing; sensitizes to chemotherapy	Preclinical	[103]
TRP Channel	TRPM7	Ca^2+^/Mg^2+^	Waixenicin A	Glioma CSCs	↓ CSC maintenance; limited use due to non-specificity	Marine natural product; preclinical	[33]
VGCC Subunit	CACNG4	Ca^2+^	Amlodipine	Hepatocellular carcinoma CSCs	↓ Stemness characteristics	FDA-approved (antihypertensive); preclinical in CSCs	[36]
Voltage-Gated K^+^ Channel	Kv1.3, others	K^+^	Margatoxin (MgTX), 4-AP	Prostate and lung CSCs	↓ Metastasis, ↑ apoptosis, G1-S arrest, ↓ lung CSC growth	MgTX preclinical; 4-AP FDA-approved (MS)	[104]
Chloride Channels	CLIC1, CLCN3	Cl^−^	DIDS, Metformin, Q48, Q54	Glioblastoma CSCs	↑ Apoptosis, ↓ proliferation, invasion, self-renewal; overcome BCNU resistance	Metformin FDA-approved (T2D); others preclinical	[48,105]
Acid-Sensing Ion Channel	ASIC1a	H^+^	PcTx1	Glioblastoma CSCs	Induces necroptosis via RIPK1	Preclinical peptide toxin	[60]
Acid-Sensing Ion Channel	ASIC3	H^+^	ASIC3 inhibitors	Glioblastoma CSCs	↓ Proliferation, migration, tumour growth	Preclinical	[73]
Acid-Sensing Ion Channel	ASIC1a	H^+^	—	Breast, prostate, pancreatic CSCs	Promotes ROS, EMT via RhoA; activates AKT/NF-κB	Mechanistic insight; no inhibitor used	[106]
Aquaporin Water Channel	AQP3	H_2_O, Glycerol	AQP3 inhibition	Gastric CSCs	↓ Self-renewal; blocks Wnt/GSK3β/β-catenin pathway	Preclinical	[107]
Aquaporin Water Channel	AQP5	H_2_O	AQP5 inhibition	Gastric CSCs	↓ Autophagy, ↓ stemness	Preclinical	[108]
Aquaporin Water Channel	AQP9	H_2_O	AQP9 restoration	Liver CSCs	↑ ROS, ↓ β-catenin activity; interacts with FOXO3a; ↓ stemness	Preclinical	[80]
Mixed Ion Channels	ENaC (Na^+^), GABA (Cl^−^), iGluRs (Na^+^, Ca^2+^)	Na^+^, Cl^−^, Ca^2+^	TTX, TEA, 4-AP, CPP, CNQX, ω-Conotoxin MVIIC, CdCl_2_	Glioblastoma CSCs	↓ CSC viability; targets enriched ion channel families	TTX, CNQX, ω-CTX, CPP are preclinical; 4-AP FDA-a	[109]

Abbreviations: CSC—cancer stem cell; VGCC—voltage-gated calcium channel; SOCE—store-operated calcium entry; MDR—multidrug resistance; GBM—glioblastoma multiforme; EMT—epithelial-to-mesenchymal transition; ROS—reactive oxygen species; RIPK1—receptor-interacting serine/threonine-protein kinase 1; FDA—Food and Drug Administration; T2D—type 2 diabetes. The downward arrow indicates a reduction, while the upward arrow denotes an increase in various parameters such as sphere formation, cell proliferation, and others.
